# Supplementary material for: Visualization and prediction of CRISPR incidence in microbial trait-space to identify drivers of antiviral immune strategy
Source: ISME J. 2019 Jun 25;13(10):2589–602. doi: 10.1038/s41396-019-0411-2 (PMC6776019; doi:10.1038/s41396-019-0411-2)
Supplement: Supplementary file 1 — Supplemental material [file 41396_2019_411_MOESM1_ESM.pdf]

Supplement - Visualization and prediction of CRISPR incidence in microbial trait-space to identify drivers of antiviral immune strategy

JL Weissman, Rohan M. R. Laljani, William F. Fagan, Philip L. F. Johnson

S1 Text Additional Models and Phylogenetic Corrections

In addition to the RF models described in the main text, we built several other models (described in Methods). Here we discuss their performance. For the logistic regression models, taking phylogeny into consideration, both via blocked cross validation (CV) ($\kappa = 0.168$) and an explicit evolutionary model of trait evolution ($\kappa = 0.188$), improved predictive ability relative to the phylogenetically-uninformed models. When combined these two corrections appeared to conflict with one another ($\kappa = 0.160$). This is to be expected based on the different ways these two approaches deal with the problem of shared evolutionary history. Blocked cross validation prevents overfitting to the underlying tree by leaving out contiguous portions of the data during the fitting process (see Methods). Phylogenetic logistic regression assumes an explicit model of trait evolution and attempts to fit that model to the data using a provided phylogeny. Because blocked CV leaves out chunks of the tree, phylogenetic logistic regression is unable to fit to those missing pieces of the tree, and thus the method's performance is reduced. In other words, blocked CV and phylogenetic logistic regression can both improve model performance when working with phylogenetically structured data, but combining these two approaches is unlikely to work well.

Moving on to our partial-least squares models (see Methods), sPLS-DA performed better than all logistic regression models, indicating that multicollinearity was likely a significant hurdle for logistic regression with subset selection (even more so than phylogenetic structure). Our cluster-based approach to phylogenetic correction (MINT) for sPLS-DA reduced overall predictive ability, but dramatically improved the true positive rate of the prediction (TPR = 0.538), at the cost of an increased false positive rate. In general there is always a tradeoff between false positive and false negative rates, but it is unclear to us why MINT sPLS-DA set its threshold for detection so low in this case. This is possibly an artefact from the differences in CRISPR incidence between our training and test sets, where MINT sPLS-DA learned to predict CRISPR presence at too low a threshold due to an overly CRISPR-enriched training set.

The RF and phylogenetically-informed RF ensemble models had nearly identical performance. We note though, that the ensemble approach gave a much more reliable estimate of predictive ability on the training set (mean $\kappa = 0.258$ predicting on excluded clusters) than the internal estimate automatically generated by the global RF model (out-of-bag estimate, $\kappa = 0.441$). In general, with phylogenetically structured data the internal error estimates generated by an RF model will be misleading, and the blocked cross-validation approach we employ is one way to correct these estimates.

S2 Text Resampling Genomes

For our main analysis we sampled one genome from RefSeq per species to assess CRISPR incidence, with a preference for completely assembled reference and representative genomes. Often, CRISPR is lost by members of a species [1], and incidence can vary among strains [2]. Therefore, we attempted to determine the potential effects of our sampling process. In general, it is better to sample single genomes to assess CRISPR incidence rather than averaging across all genomes for a given species, since species are unevenly represented in RefSeq and thus the variances in incidence between species will not be equal. A drawback of sampling is that it throws away information, although strong trends should still be apparent if species have consistent tendencies to either possess or lack CRISPR. In fact, for 84% of the species in our trait dataset, the available genomes either all have or all lack CRISPR (no within species variability). Thus, sampling should have relatively minor effects on our outcome.

We verified this by repeatedly resampling CRISPR incidences from the set of all RefSeq genomes (previously determined in [3]). First we randomly resampled a new genome with known CRISPR incidence for each species in the dataset, then we split the data into training and test sets (using Proteobacteria again as the test set) and built an RF model as in the main text. This process was repeated 1000 times, and the resulting κ values and top predictors are reported in S21 Fig. The results of this analysis were consistent with our analysis in the main text.

S3 Text CRISPR in the Tara Oceans Data

An alternative, and complementary approach to the one we took here is to directly measure the change in prevalence of a particular immune strategy (e.g. CRISPR) across environmental gradients using metagenomics. This approach has its own pitfalls and will require its own solutions. For example, in complex communities it may be difficult to link CRISPR proteins to particular genomes or organisms, meaning that it will be difficult to differentiate between changes in CRISPR prevalence due to differential gene content in the same set of organisms and changes in prevalence due to shifts in community composition. The situation is further complicated by the fact that many organisms have multiple CRISPR systems, or conversely have partial and non-functional systems, and that CRISPR and other defense systems are extremely labile, being gained and lost frequently [1, 3, 4]. This makes the metagenome assembly process significantly more difficult with respect to correctly mapping CRISPR to host. We also note that our current dataset integrates microbial traits across many scales, whereas a metagenomic approach will only link CRISPR prevalence to the coarsest scale of environmental parameters. Even considering oxygen, in many environments there is a possibility for extremely fine-grained variation that allows aerobes and anaerobes to live in close proximity (e.g. anoxic sediments in wetlands aerated by plant roots). In other words, our approach in the main text labels microbes as “is this”, whereas relating environmental gradients to metagenomic data labels microbes as “lives here”, where “here” is by necessity an average across the sample. A metagenomics approach links immune strategy to microbial traits indirectly via environment.

Nevertheless, metagenomics is an attractive alternative because it allows us to analyze strategy shifts actually occurring in the environment. While it is beyond the scope of the current study to perform extensive analyses of metagenomic datasets, we wish to provide an encouraging example to motivate future work in what we think is an exciting area. We used data from the Tara Oceans project [5], a global study of the microbial communities in earth’s oceans, as our case study. The

dataset consisted of a set of functional profiles provided by Tara, in which reads were mapped to particular orthologous groups (OG) using the KEGG orthologous groups database, as well as environmental metadata for each sample [5]. We identified the OGs for *cas1* and *cas2* (universal CRISPR marker genes; K15342 and K09951), *cas3* (type I marker; K070012), *cas9* (type II; K09952), and *cas10* (type III; K07016). We then normalized the coverage of each OG by total coverage in a given sample, and paired this data with the dissolved oxygen concentration for each sample.

Similar to our results based on ProTraits, we found a negative association between oxygen and CRISPR (S22 Fig). We found a significant negative correlation between oxygen and *cas1* (Pearson's product moment correlation, $\rho = -0.1757433$, $p = 0.00668$), *cas2* ($\rho = -0.2254487$, $p = 0.0004696$), *cas3* ($\rho = -0.1939399$, $p = 0.002714$), and *cas10* ($\rho = -0.4018567$, $p = 1.304 \times 10^{-10}$). The relationship between oxygen and *cas9* was not significant ($\rho = -0.03446256$, $p = 0.5976$). We note that this data doesn't strictly represent an oxygen "gradient" since dissolved oxygen content appears to be bimodal, with peaks corresponding to oxic and anoxic conditions (S22 Fig).

S4 Text Number of CRISPR Arrays

Many prokaryotes have multiple CRISPR arrays, and this multiplicity is potentially maintained by selection [3]. We sought to assess whether we could predict the multiplicity of CRISPR arrays on a genome using our trait data. CRISPRDetect identifies individual arrays, so that our original dataset already included information about array multiplicity as well as incidence. We excluded all species lacking CRISPR so as not to confound the question of incidence (who has CRISPR?) with multiplicity (how many CRISPR arrays do they have?). Random forests can be used on continuous outcome variables (regression), and so we built a RF model using the same procedure as in the main text, but with multiplicity rather than incidence as the outcome variable. This model performs extremely poorly, with essentially no predictive ability (MSE = 4.26, $R^2 = 0.008$). The predicted and actual values on the test set were barely significantly correlated (Pearson's correlation, $\rho = 0.09$, $p = 0.048$). This is not entirely surprising, as regression is generally more difficult that classification. In other words, it is harder to predict whether an organism has one, two, or three CRISPR arrays than it is to predict if it has CRISPR at all.

S5 Text NHEJ-Oxygen Model

Using our annotations for Ku and the NCBI annotations for oxygen requirement (aerobes and anaerobes only, facultative organisms excluded) we compiled a set of 1473 genomes for which both pieces of information were available. We built a phylogeny for these genomes using the method described in the main text. We then built a phylogenetically corrected linear model with CRISPR incidence as the binary outcome variable, Ku and aerobicity as binary predictors, as well as an interaction term (phylogenetic logistic regression, phylolm R package; [6, 7]). Ives and Garland [6] recommend that when categories have small sample sizes (as does our anaerobe with Ku category at 33 genomes) that *p*-values for phylogenetic logistic regression are obtained via bootstrapping, although this method is more computationally intensive. We performed 1000 bootstrap replicates (the 'boot' option in the phyloglm() function) to assess the statistical significance of each term in the model. We repeated this analysis with the *cas3*, *cas9*, and *cas10* genes, which are diagnostic of CRISPR system type, in order to see if any Ku-oxygen-CRISPR interaction was type-specific.

Our bootstrapped p values for both Ku and Oxygen, as well as their interaction, were all below 0.001 (all bootstrapped coefficients differed from zero in a consistent direction across all replicates). These p-values differed from the maximum likelihood estimates generated from the phylogenetic logistic regression model (notably, the interaction between Ku and oxygen was not significant using these estimates, at $p = 0.088164$, though the effects of Ku 1.53×10^{-5}, and oxygen 0.001183 remained significant), though this should not be surprising as the behavior of these estimates are not well characterized at low sample sizes and bootstrap estimates are generally the favored approach [6].

For type I and III systems, the results were generally consistent. In the case of type I systems all model terms were significant under bootstrapping (Ku $p < 0.001$, oxygen $p = 0.016$, interaction $p < 0.001$) but when using p-values from the ML estimate oxygen was not a significant predictor of *cas3* incidence (Ku $p = 4.164 \times 10^{-10}$, oxygen $p = 0.1138959$, interaction $p = 0.0004477$). The same was true for type III systems in terms of bootstrapped p-values (Ku $p < 0.001$, oxygen $p = 0.039$, interaction $p = 0.002$) and those from the ML estimates (Ku $p = 0.0002035$, oxygen $p = 0.3048236$, interaction $p = 0.0014942$). For type II systems only the effects of Ku were significant, and only in the bootstrapped case (Ku $p = 0.005$, oxygen $p = 0.052$, interaction $p = 0.0546$), not for the ML estimates (Ku $p = 0.1176$, oxygen $p = 0.2542$, interaction $p = 0.7550$).

For all of these phylogenetic regressions, results were consistent on 10 bootstrapped trees (S4 Table).

S6 Text ProTraits Without Genomic Data

The ProTraits database, from which we take our trait data, combines various "sources" of text-based and genomic information to make trait predictions [8]. While the inclusion of genomic sources of information considerably improves the trait confidence scores, some of these sources explicitly consider gene presence/absence, and we worried it may lead to circularity in our arguments (e.g. if *cas* gene presence were used to predict a trait, which was then used to predict CRISPR incidence). Therefore we repeated our predictive analyses excluding the "phyletic profile" and "gene neighborhood" sources in ProTraits. We took the maximum confidence scores for having and lacking a trait respectively across all other sources in the database to produce a negative and positive trait score. We integrated these into a single score as described in Methods. We then built an RF model of CRISPR incidence, as this was the highest performing model on the complete dataset. This model had comparable predictive ability ($\kappa = 0.243$). We also found similar predictors to when the full dataset was used (S9 Fig). A notable change is that termite host and PAH degradation no longer appear as important predictors in the model.

S7 Text Outline of Analyses

Visualizations

- CRISPR Incidence
  - PCA (Fig 1, Table 1, S2 Fig)
  - t-SNE (Fig 2, S3 Fig, S17 Fig)
- CRISPR Type

    - PCA (Fig 5)

- RM Incidence
    - PCA (S13 Fig)

- Ku Incidence
    - PCA (S10 Fig)

Predictive Models (Proteobacteria Test Set)

Comparison of all predictive models in S1 Fig

- CRISPR Incidence (Table 2, S1 Text, S1 Fig)
    - Logistic Regression with Forward Subset Selection and Random CV (S1 Table)
    - Logistic Regression with Forward Subset Selection and Blocked CV (S1 Table)
    - Phylogenetic Logistic Regression with Forward Subset Selection and Random CV (S1 Table)
    - Phylogenetic Logistic Regression with Forward Subset Selection and Blocked CV (S1 Table)
    - sPLS-DA (S4 Fig)
    - MINT sPLS-DA (S5 Fig)
    - Random Forest (Fig 3, S7 Fig)
    - Random Forest Ensemble (S6 Fig)
    - Random Forest, no genetic information (S6 Text, S9 Fig)

- CRISPR Incidence With only Temperature and Oxygen as Predictors to Train Model
    - Random Forest

- Number of CRISPR Systems
    - Random Forest (regression; S4 Text)

- Type II CRISPR Incidence (*cas9*)
    - Random Forest (Fig 5)

- RM Incidence
    - Random Forest (S14 Fig)

- Ku Incidence
    - Random Forest (S11 Fig)

Phylogenetic Regressions

- CRISPR vs 16s rRNA count (also on bootstrapped trees; S2 Table)
- CRISPR vs Ku and Oxygen (also on bootstrapped trees, oxygen use from NCBI metadata; S5 Text, S4 Table)
- Number of Restriction Enzymes vs Temperature (also on bootstrapped trees; S3 Table)
- Number of Restriction Enzymes vs Oxygen (also on bootstrapped trees; S3 Table)

Metagenomic Data

- *cas1,2,3,9,10* Coverage vs Dissolved Oxygen (S3 Text, S22 Fig)

Other

- CRISPR vs Temperature and Oxygen (NCBI metadata)
  - Binomial confidence intervals (Fig 4, S8 Fig)
  - χ^2 test
- Correlation between CRISPR and Ku
- Resampling genomes for CRISPR incidence (S2 Text, S21 Fig)

References

[1] Jiang W, Maniv I, Arain F, Wang Y, Levin BR, Marraffini LA. Dealing with the Evolutionary Downside of CRISPR Immunity: Bacteria and Beneficial Plasmids. PLOS Genetics. 2013 Sep;9(9):e1003844.

[2] Westra ER, Dowling AJ, Broniewski JM, Houte Sv. Evolution and Ecology of CRISPR. Annual Review of Ecology, Evolution, and Systematics. 2016;47(1):307–331.

[3] Weissman JL, Fagan WF, Johnson PL. Selective maintenance of multiple CRISPR arrays across prokaryotes. The CRISPR Journal. 2018;1(6):405–413.

[4] Puigbò P, Makarova KS, Kristensen DM, Wolf YI, Koonin EV. Reconstruction of the evolution of microbial defense systems. BMC Evolutionary Biology. 2017 Apr;17:94.

[5] Sunagawa S, Coelho LP, Chaffron S, Kultima JR, Labadie K, Salazar G, et al. Structure and function of the global ocean microbiome. Science. 2015;348(6237):1261359.

[6] Ives AR, Garland T. Phylogenetic Logistic Regression for Binary Dependent Variables. Systematic Biology. 2010 Jan;59(1):9–26.

[7] Ho LsT, Ané C. A linear-time algorithm for Gaussian and non-Gaussian trait evolution models. Systematic Biology. 2014 May;63(3):397–408.

[8] Brbić M, Piškorec M, Vidulin V, Kriško A, Šmuc T, Supek F. The landscape of microbial phenotypic traits and associated genes. Nucleic Acids Research. 2016 Dec;44(21):10074–10090.

Logistic Regression	
Random CV	Blocked CV
temperaturerange_thermophilic (+)	temperaturerange_thermophilic (+)
mammalian_pathogen_oral_cavity (+)	mammalian_pathogen_oral_cavity (+)
knownhabitats_freshwater (+)	metabolism_carbondioxidefixation (+)
ecosystemtype_marine (-)	host_insectstermites (+)
pathogenic_in_mammals (-)	ecosystemtype_geologic (+)
knownhabitats_hydrothermalvent (+)	energysource_autotroph (+)
metabolism_carondioxidefixation (+)	ecosystemsubtype_vagina (+)
host_insectstermites (+)	metabolism_sulfuroxidizer (-)
shape_tailed (+)	habitat_terrestrial (-)
knownhabitats_soil (-)	
knownhabitats_creosotecontaminatedsoil (-)	
energysource_heterotroph (-)	
cellarrangement_tetrads (-)	
ecosystemsubtype_vagina (+)	
knownhabitats_insectendosymbiont (-)	
ecosystemtype_thermalsprings (+)	
habitat_hostassociated (+)	
cellarrangement_singles (-)	

Phylogenetic Logistic Regression	
Random CV	Blocked CV
knownhabitats_hotspring (+)	knownhabitats_hotspring (+)
mammalian_pathogen_oral_cavity (+)	mammalian_pathogen_oral_cavity (+)
host_insectstermites (+)	host_insectstermites (+)
shape_filamentous (+)	shape_filamentous (+)
oxygenreq_strictaero (-)	oxygenreq_strictaero (-)
ecosystemtype_reproductivesystem (+)	energysource_heterotroph (-)
mammalian_pathogen_respiratory_lundisease (-)	
ecosystemtype_marine (-)	
knownhabitats_hydrothermalvent (+)	
ecosystemcategory_plants (-)	

S1 Table: Predictors added to each logistic regression model during forward selection (top to bottom in order of addition). Plus and minus signs indicate whether a variable is positively or negatively associated with CRISPR incidence.

S2 Table: Phylogenetic logistic regression of CRISPR incidence as predicted by 16s rRNA count on 10 bootstrapped trees.

Outcome Variable	Bootstrap	β_{16s}	p_{16s}
CRISPR	1	0.05444265	0.0004987372
CRISPR	2	0.06871256	1.650863E-05
CRISPR	3	0.05602856	0.0003348601
CRISPR	4	-0.06244074	4.65824E-05
CRISPR	5	-0.06051718	7.066252E-05
CRISPR	6	-0.0656118	1.96243E-05
CRISPR	7	-0.06858516	9.275297E-06
CRISPR	8	-0.06523327	2.200228E-05
CRISPR	9	-0.06482068	2.414822E-05
CRISPR	10	0.06773283	1.521424E-05

S3 Table: Phylogenetic regression of number of restriction enzymes as predicted by temperature or oxygen on 10 boostrapped trees.

Outcome Variable	Bootstrap	$\beta_{Temperature}$	$p_{Temperature}$
No. R Enzymes	1	1.46992099	0.04000844
No. R Enzymes	2	1.47619604	0.0395859
No. R Enzymes	3	0.05938679	0.67987639
No. R Enzymes	4	1.47619604	0.0395859
No. R Enzymes	5	1.49642946	0.03825504
No. R Enzymes	6	1.46992099	0.04000844
No. R Enzymes	7	1.46196112	0.04055124
No. R Enzymes	8	1.51134694	0.0373039
No. R Enzymes	9	0.0593766	0.67990236
No. R Enzymes	10	1.51134694	0.0373039
Outcome Variable	Bootstrap	β_{O^2}	p_{O^2}
No. R Enzymes	1	-4.5032905	4.775284E-35
No. R Enzymes	2	-4.5236085	3.343288E-35
No. R Enzymes	3	-0.9838951	1.133434E-08
No. R Enzymes	4	-4.5262046	3.194396E-35
No. R Enzymes	5	-4.540566	2.482726E-35
No. R Enzymes	6	-4.5216758	3.458622E-35
No. R Enzymes	7	-4.5195994	3.586961E-35
No. R Enzymes	8	-4.5446124	2.312513E-35
No. R Enzymes	9	-0.9838037	1.135213E-08
No. R Enzymes	10	-4.5419952	2.421222E-35

S4 Table: Phylogenetic logistic regression of CRISPR incidence as predicted by Ku and oxygen on 10 boostrapped trees. Bootstrapped p-values shown as discussed in S5 Text

Outcome Variable	Bootstrap	β_{Ku}	p_{Ku}	β_{O^2}	p_{O^2}	$\beta_{\text{Interaction}}$	$p_{\text{Interaction}}$
CRISPR	1	-0.7776371	0.001	0.66532	0.001	0.8076997	0.001
CRISPR	2	-0.7762393	0.001	0.6650369	0.001	0.7553189	0.001
CRISPR	3	-0.7543548	0.001	0.6523729	0.001	0.7743154	0.002
CRISPR	4	-0.7475297	0.001	0.6470698	0.001	0.7970853	0.001
CRISPR	5	-0.7542887	0.001	0.6499091	0.001	0.7606847	0.001
CRISPR	6	-0.7750393	0.001	0.6560871	0.001	0.7523748	0.001
CRISPR	7	-0.752069	0.001	0.6479241	0.001	0.8017921	0.001
CRISPR	8	-0.7570104	0.001	0.6461722	0.001	0.7978345	0.001
CRISPR	9	-0.7931716	0.001	0.676406	0.001	0.7285855	0.001
CRISPR	10	-0.7782145	0.001	0.6634043	0.001	0.7725446	0.002
cas3	1	-1.309984	0.001	0.3429482	0.009	1.586328	0.001
cas3	2	-1.339081	0.001	0.3289939	0.007	1.582142	0.001
cas3	3	-1.304121	0.001	0.3257619	0.017	1.581483	0.001
cas3	4	-1.311048	0.001	0.2938582	0.011	1.590015	0.001
cas3	5	-1.333989	0.001	0.3291178	0.023	1.586987	0.001
cas3	6	-1.315037	0.001	0.3253995	0.008	1.585864	0.001
cas3	7	-1.308	0.001	0.2901924	0.014	1.58649	0.001
cas3	8	-1.325072	0.001	0.3139974	0.023	1.59048	0.001
cas3	9	-1.351763	0.001	0.3449322	0.007	1.590153	0.001
cas3	10	-1.328922	0.001	0.3384614	0.014	1.584191	0.001
cas9	1	-0.6166104	0.001	0.3386759	0.05	-0.3086753	0.536
cas9	2	-0.5713407	0.011	0.4218407	0.019	-0.3035605	0.538
cas9	3	-0.6371981	0.002	0.380116	0.04	-0.3139595	0.545
cas9	4	-0.2427449	0.05	1.0055809	0.025	-0.3538297	0.451
cas9	5	-0.598588	0.005	0.3807694	0.04	-0.304255	0.556
cas9	6	-0.6344803	0.006	0.4011055	0.02	-0.3178936	0.565
cas9	7	-0.6162547	0.01	0.3864871	0.017	-0.2999418	0.555
cas9	8	-0.6261813	0.002	0.3399736	0.059	-0.3096002	0.564
cas9	9	-0.6106022	0.006	0.4121876	0.011	-0.3016357	0.552
cas9	10	-0.5917813	0.003	0.381879	0.051	-0.3111323	0.523
cas10	1	-2.78319	0.001	0.338453	0.048	3.0924286	0.001
cas10	2	-2.76847	0.001	0.3371324	0.028	3.0689795	0.001
cas10	3	-0.735233	0.002	0.2797778	0.039	0.7382052	0.028
cas10	4	-1.209831	0.001	0.3202466	0.027	1.2494469	0.014
cas10	5	-2.927175	0.001	0.3800277	0.047	3.1038217	0.001
cas10	6	-2.750308	0.001	0.3269329	0.05	3.080121	0.001
cas10	7	-2.706733	0.001	0.3376516	0.032	2.9835339	0.001
cas10	8	-2.839044	0.001	0.3164233	0.064	3.116515	0.001
cas10	9	-1.944355	0.001	0.3521944	0.026	2.244925	0.002
cas10	10	-2.891364	0.001	0.3741208	0.03	3.1046525	0.001

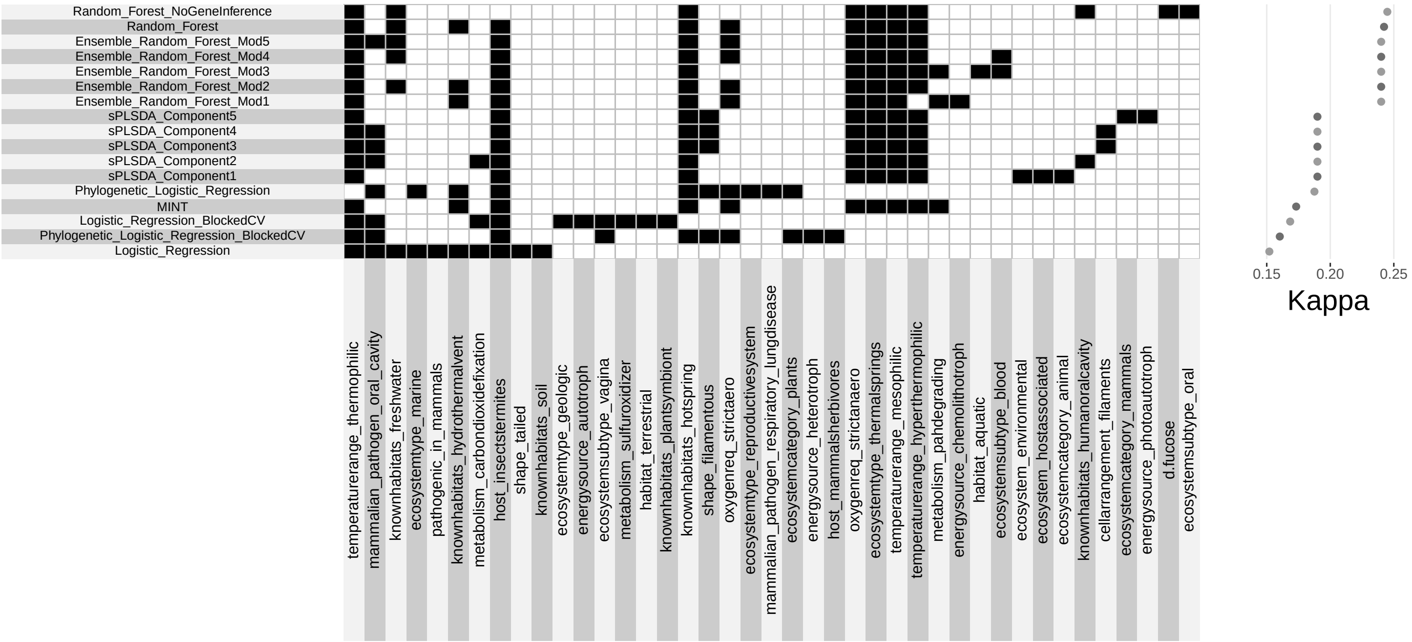

(a)

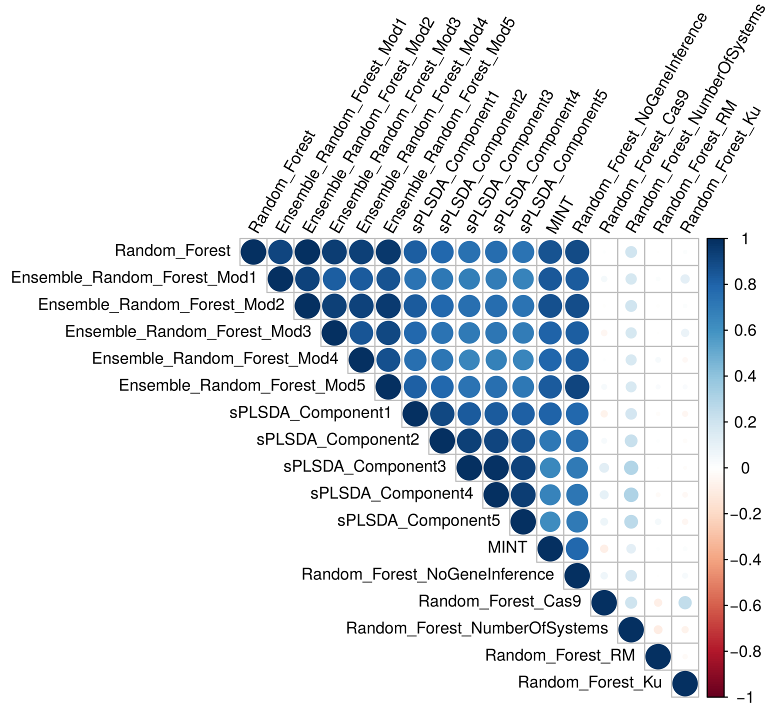

(b)

S1 Fig: Comparison of variable importance across predictive models. (a) The top 10 most important variables (columns) for each predictive model of CRISPR incidence (rows) are indicated by filled black cells. Models are ordered top to bottom in order of decreasing performance in terms of Cohen's κ (shown at right). Note that for the high performing models, temperature variables and oxygen (anaerobe and aerobe) are consistently found in the top 10 predictors. All models incorporate temperature as an important predictor, and the only models without oxygen as a top predictor are the two logistic regression models that were not formally corrected for phylogeny (and were low-performing). In general, moderate and high performing models are largely in agreement about a core set of important variables. (b) Pearson's correlation between variable importance scores for all predictive models (CRISPR incidence and otherwise) in the paper measured as % increase in node purity for random forests and variable importance projections for PLS models; logistic regression models were excluded because importance is measured as rank - i.e. what order the variable was added to the model. Note the high agreement between the models predicting CRISPR incidence, and some agreement with the model predicting number of CRISPR systems. Also note that models predicting the incidence of RM systems and Ku appear to have distinct predictors (these models performed well at prediction tasks in the main text). "NoGeneInference" corresponds to the model built in S6 Text.

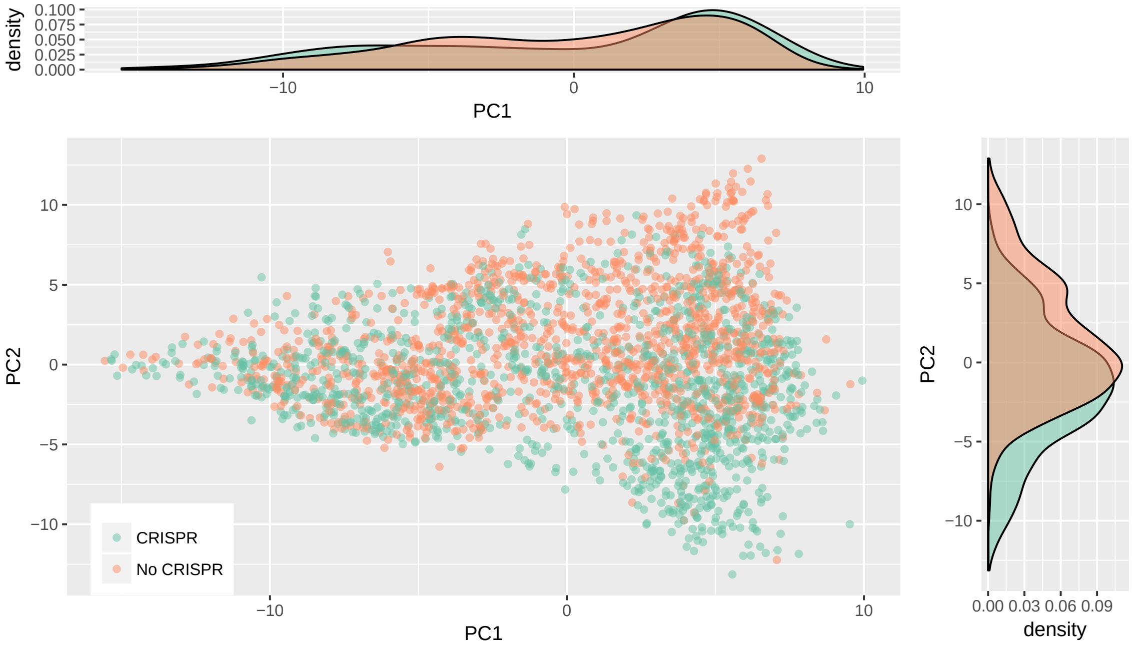

S2 Fig: Organisms with CRISPR do not separate from those without along the first principal component of trait space. The first and second components from a PCA of the microbial traits dataset are shown. CRISPR incidence is indicated by color (green with, orange without), but was not included when constructing the PCA. Marginal densities along each component are shown to facilitate interpretation. See Fig 1 for the third component.

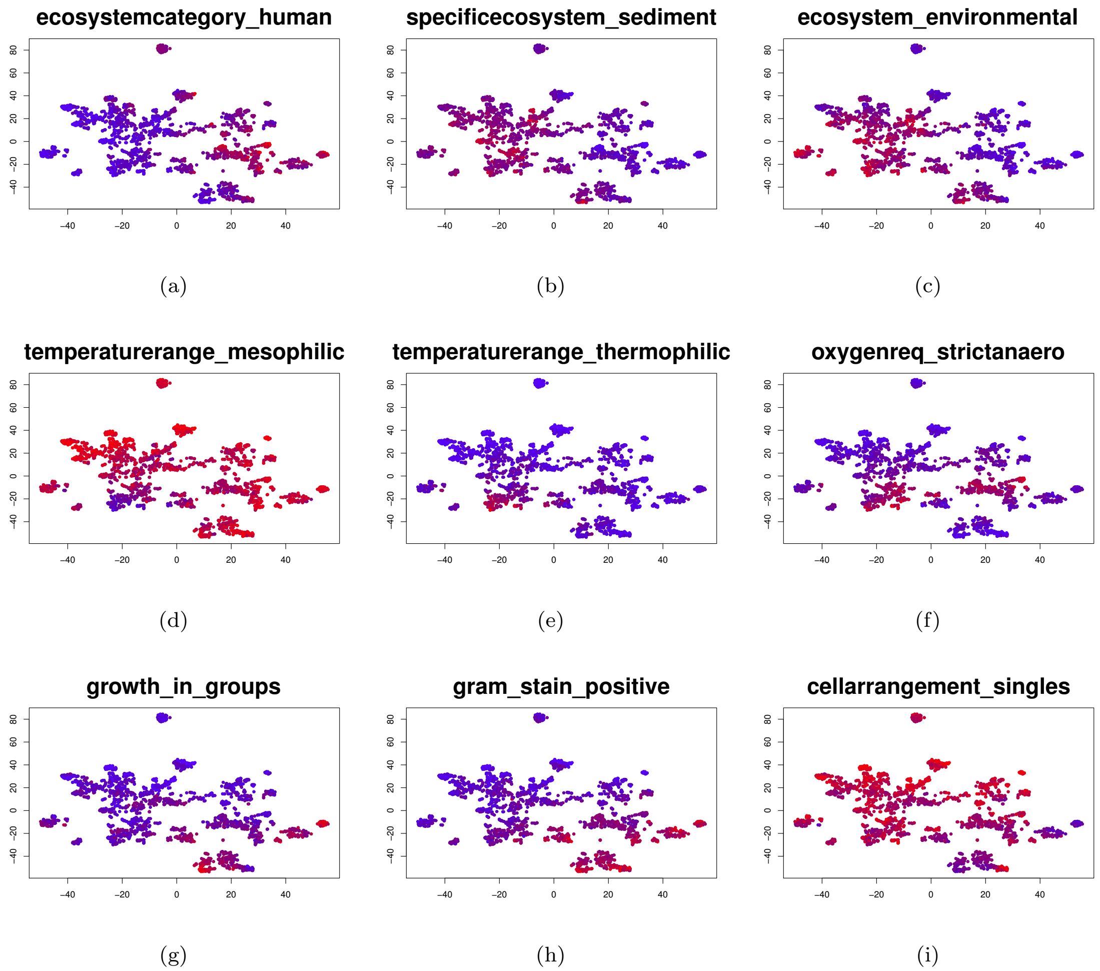

S3 Fig: Trait distributions over t-SNE reduced dataset. Each point is an organism mapped onto our t-SNE decomposition of trait space. Instead of coloring points by presence/absence of CRISPR as shown in Fig 2, we color each organism by its score for selected microbial traits in our trait dataset (set of traits shown chosen because they were highly weighted in our PCA). Recall that scores range from zero (blue) to one (red). We note that, in a general sense, the region occupied by anaerobic microbes appears to correspond to the densest regions of CRISPR incidence in Fig 2.

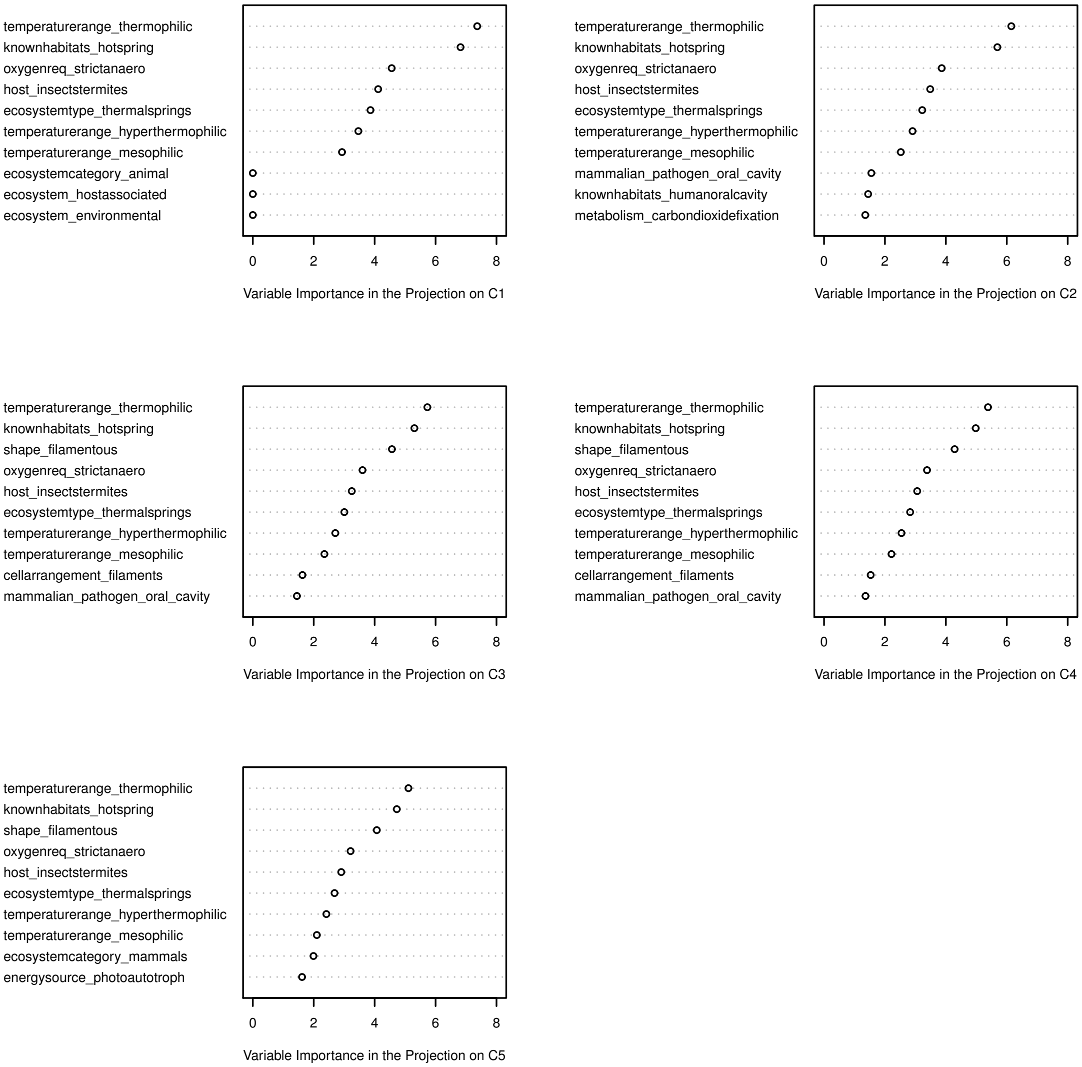

S4 Fig: Variable importance scores from sPLS-DA model for top 10 predictors on the 5 components included in model. Variable importance scores generated by the vip() function in the mixOmics package for R.

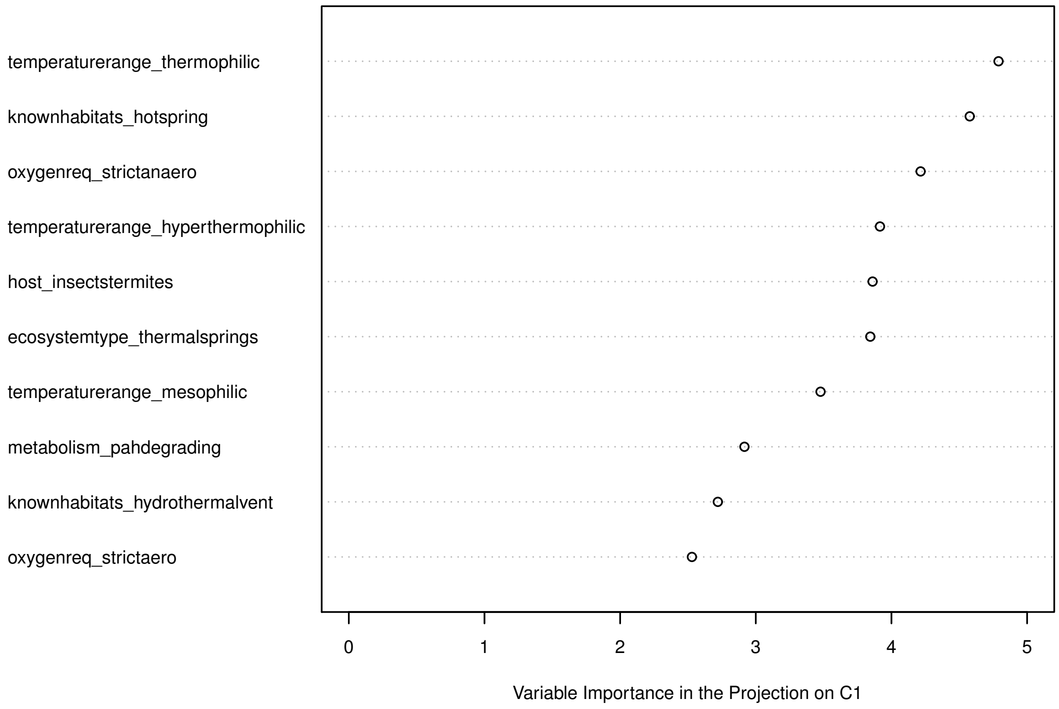

S5 Fig: Variable importance scores from MINT sPLS-DA model for top 10 predictors on the single component included in model. Variable importance scores generated by the vip() function in the mixOmics package for R.

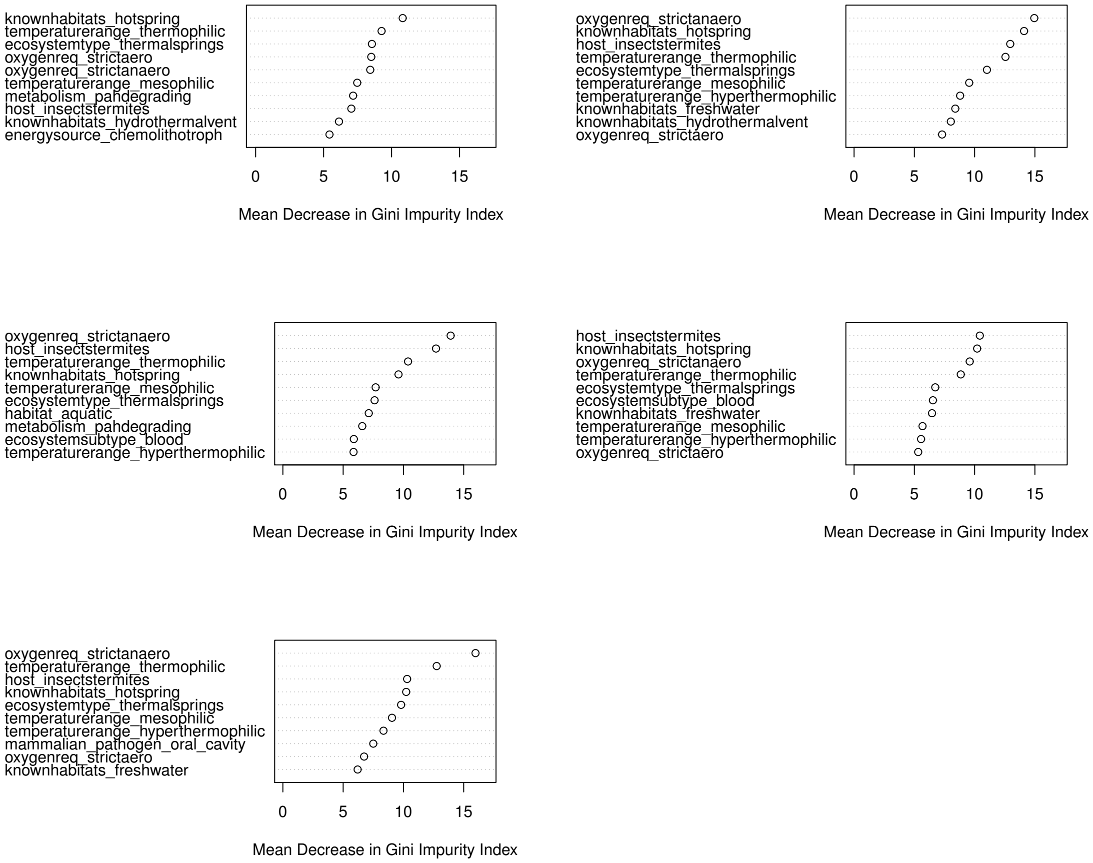

S6 Fig: Importance of top ten predictors in each of the five forests included in the RF ensemble model, as measured by the mean decrease in the Gini impurity index or accuracy when that variable is excluded from the respective forest. The relative ranking of the top ten predictors does vary somewhat over the five forests, but the set of top predictors is largely consistent across the forests.

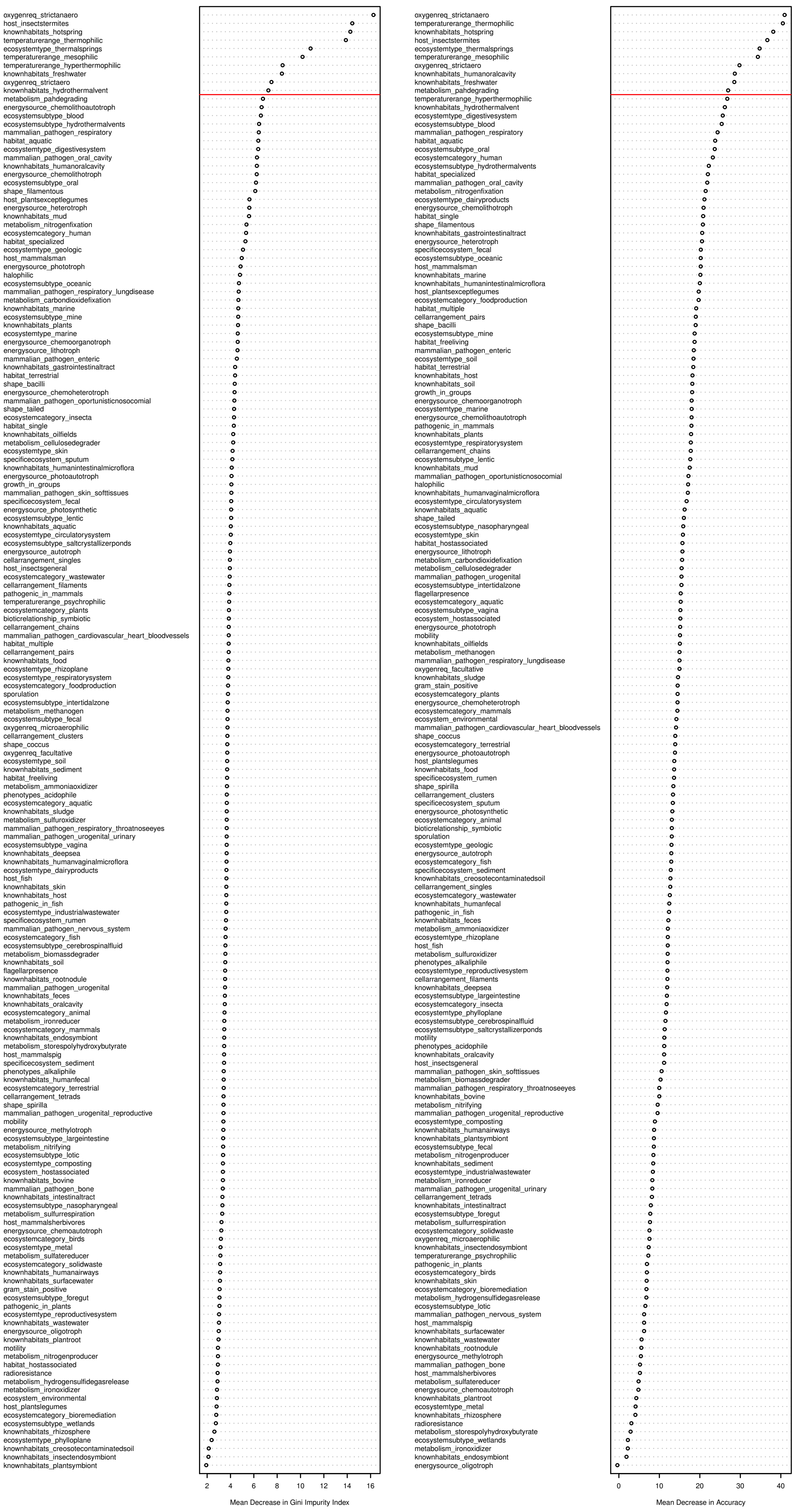

S7 Fig: Importance of all predictors in CRISPR RF model, as measured by the mean decrease in the Gini impurity index or accuracy when that variable is excluded from the respective forest. Note the elbow in the Gini importance ranking after the first ten predictors.

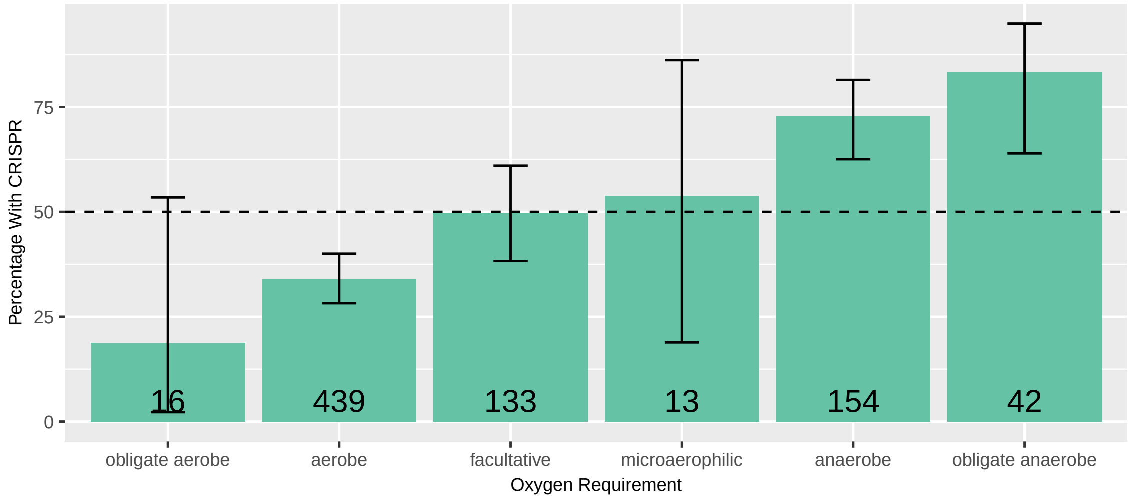

S8 Fig: The link between oxygen requirement and CRISPR incidence is apparent even when subsetting to only mesophiles. Error bars are 99% binomial confidence intervals. Total number of genomes in each trait category shown at the bottom of each bar. Categories represented by fewer than 10 genomes were omitted.

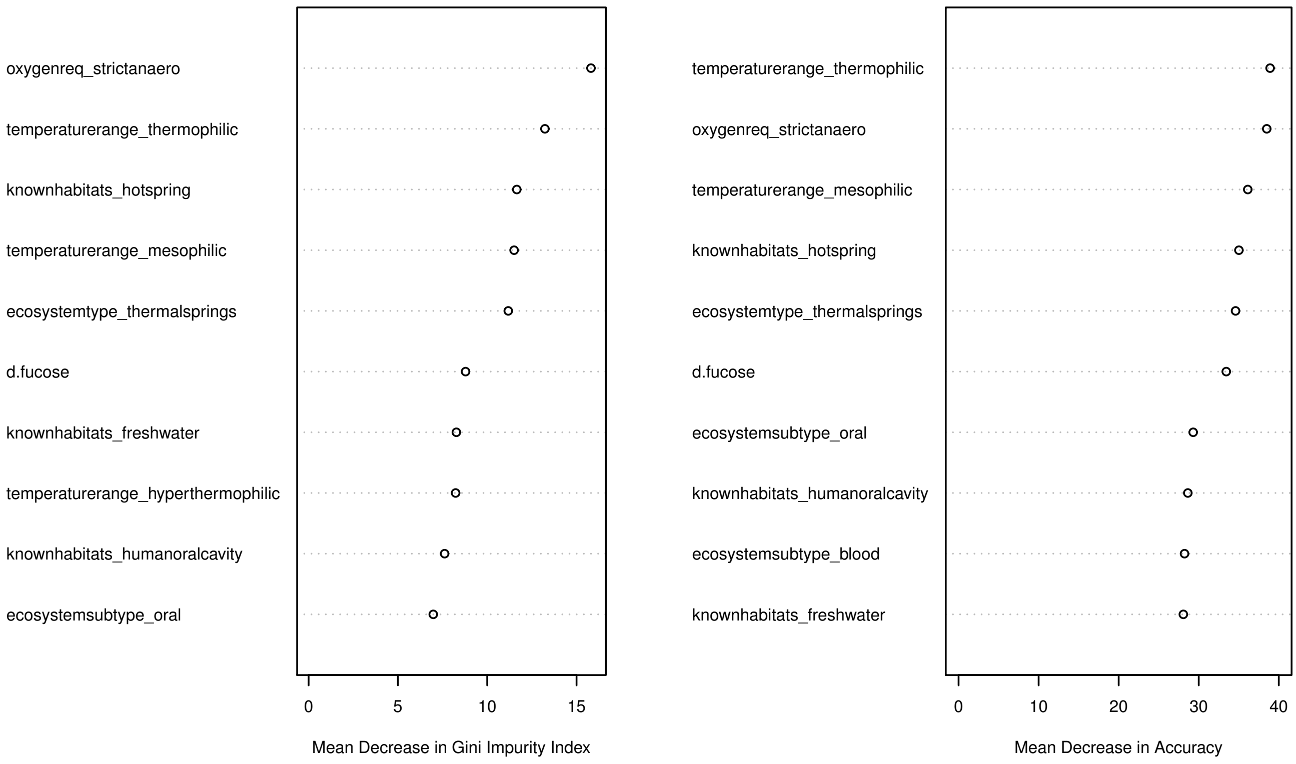

S9 Fig: Importance of top ten predictors in the RF model built excluding the "phyletic profile" and "gene neighborhood" information sources from ProTraits, as measured by the mean decrease in the Gini impurity index or accuracy when that variable is excluded from the model.

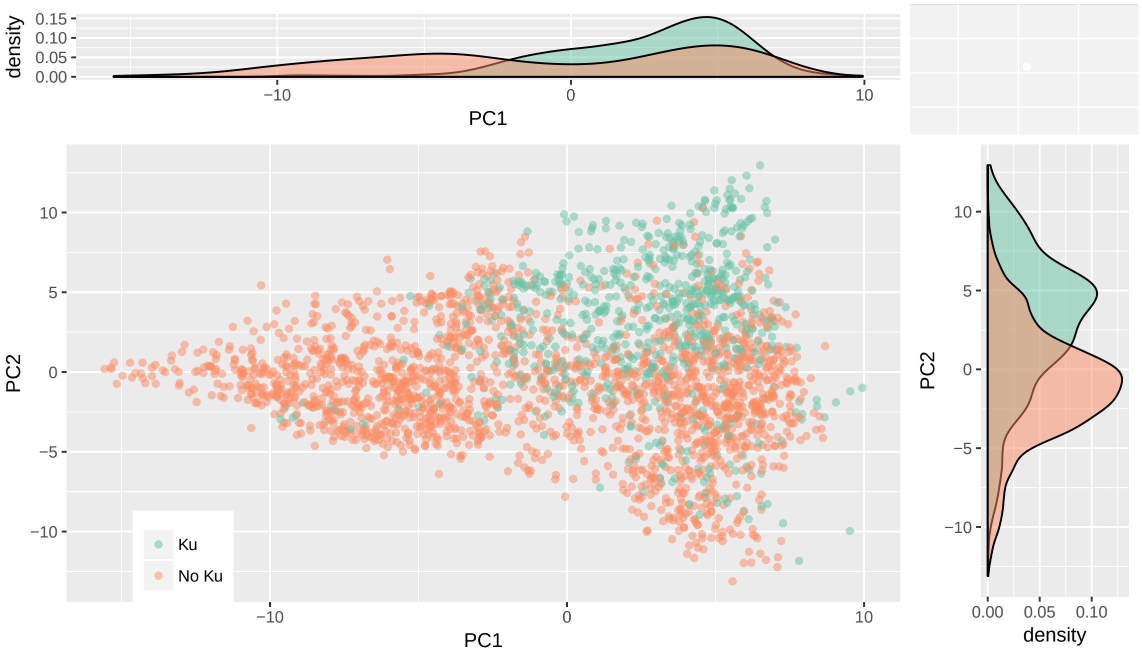

(a)

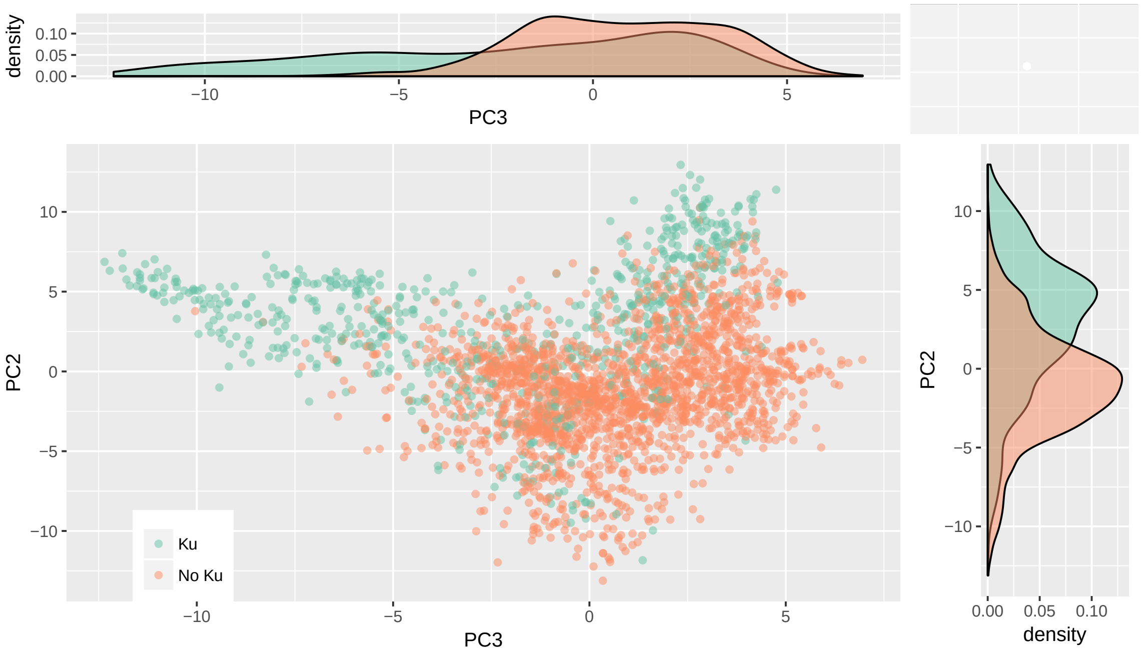

(b)

S10 Fig: The incidence of the Ku protein in trait space. PCA as in Fig 1 and S2 Fig.

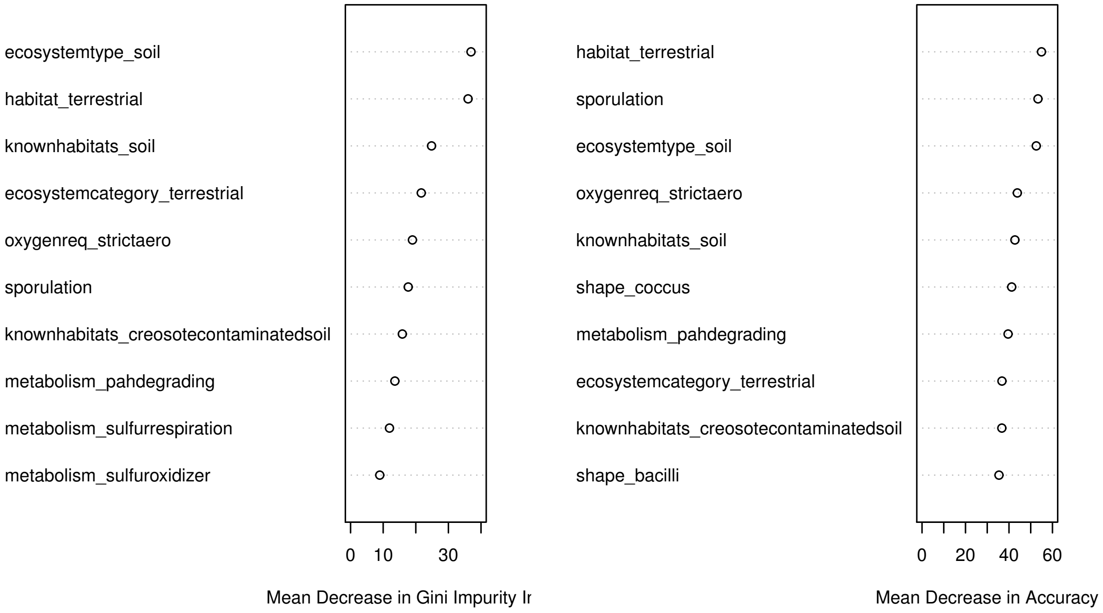

S11 Fig: Importance of top ten predictors in the RF model of Ku incidence. This model had high predictive ability ($\kappa = 0.578$).

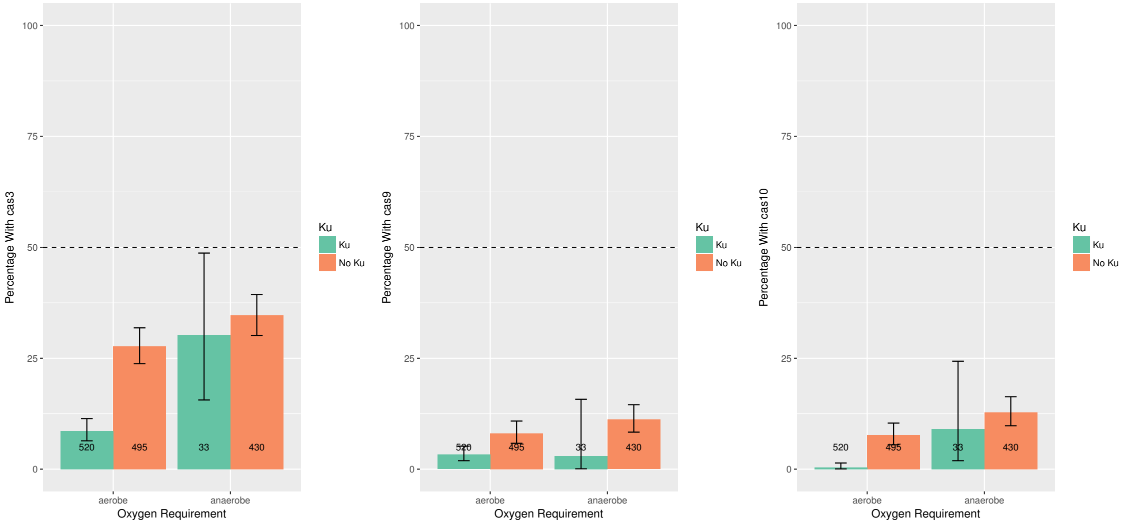

(a) (b) (c)

S12 Fig: CRISPR and Ku are negatively associated in aerobes but not anaerobes. Percentage of genomes with Cas proteins associated with a particular system type. Error bars are 99% binomial confidence intervals. Total number of genomes in each trait category shown at the bottom of each bar. Of the 1047 genomes represented here 253 have *cas3*, 61 have *cas9*, and 54 have *cas10*.

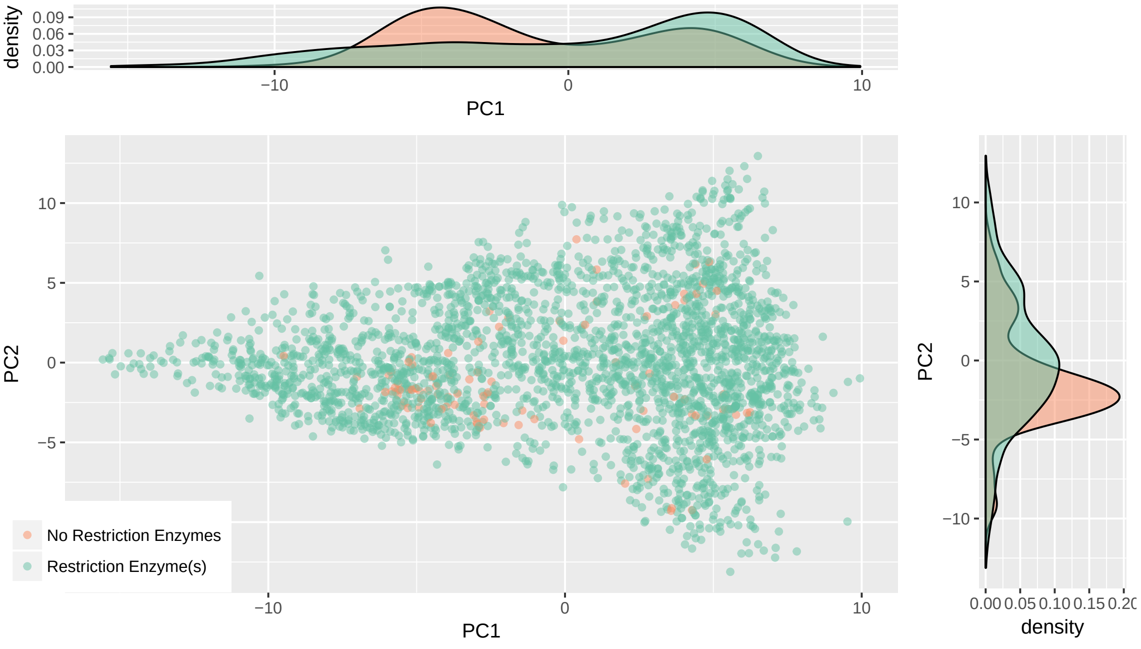

(a)

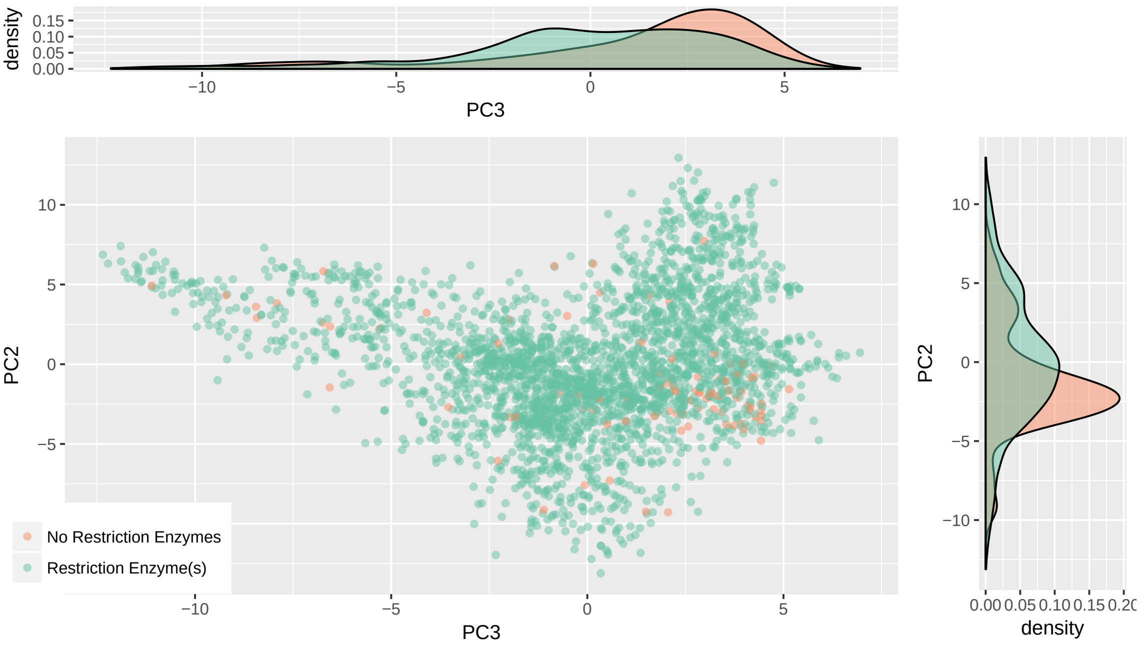

(b)

S13 Fig: The incidence of restriction enzymes in trait space. PCA as in Fig 1 and S2 Fig.

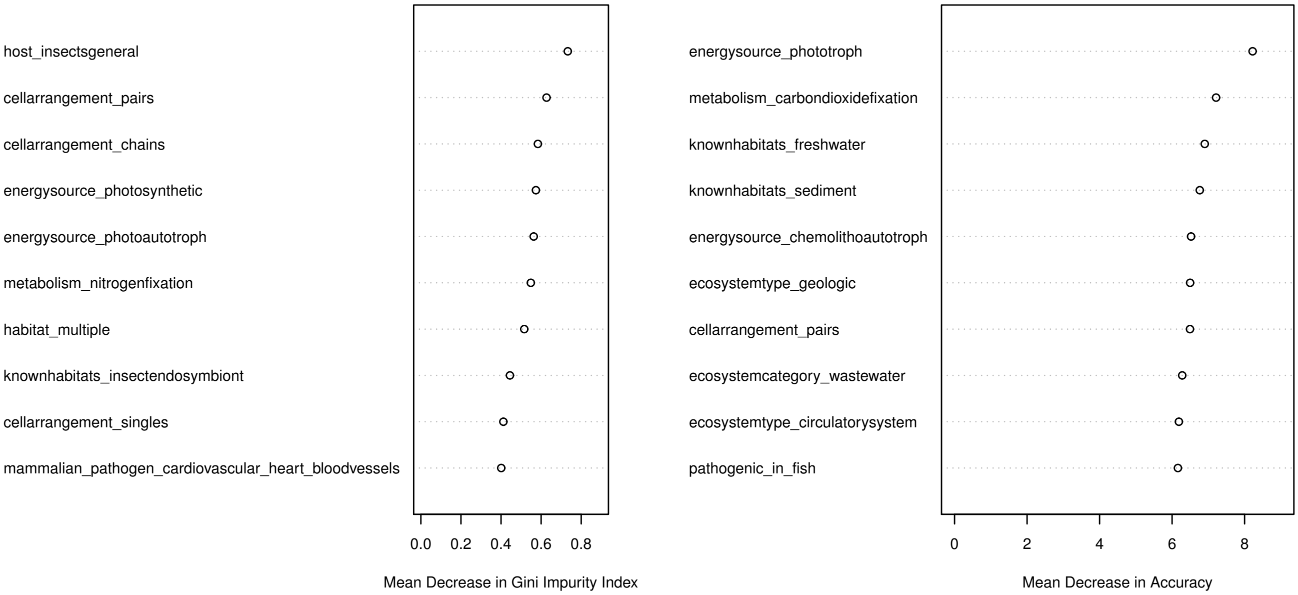

S14 Fig: Importance of top ten predictors in the RF model of restriction enzyme incidence, as measured by the mean decrease in the Gini impurity index or accuracy when that variable is excluded from the model.

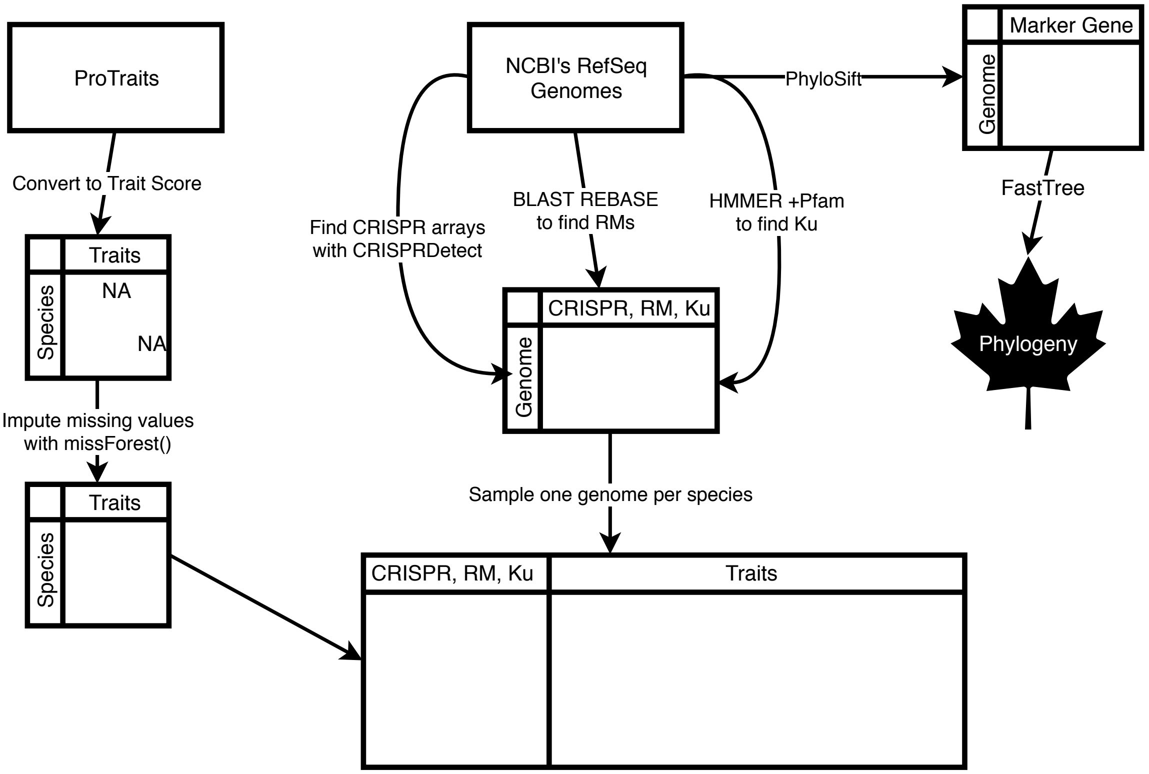

S15 Fig: Pipeline for generating trait and immunity dataset and matching phylogeny. See Methods for details.

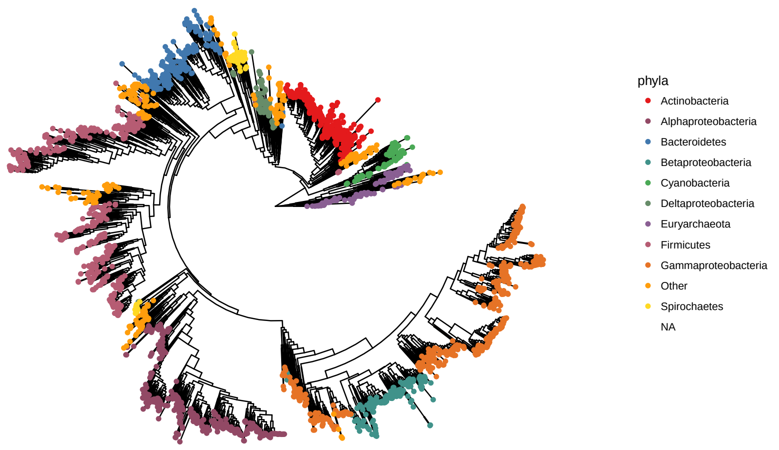

S16 Fig: Phylogeny generated from PhyloSift marker genes. Phylum indicated by color, with taxonomic classifications taken from NCBI.

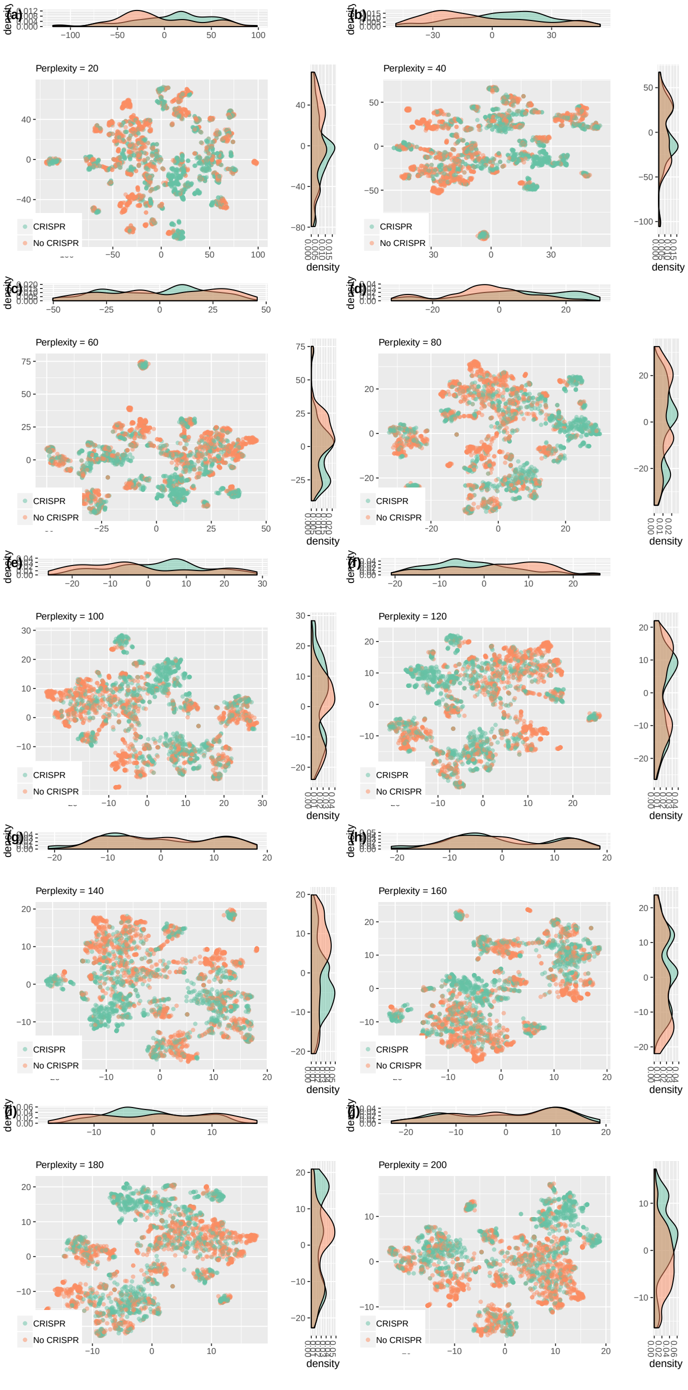

S17 Fig: Repeated t-SNE decomposition of ProTraits data with CRISPR incidence visualized for varied perplexity values. The CRISPR versus no-CRISPR separation is somewhat less apparent for very high perplexity values.

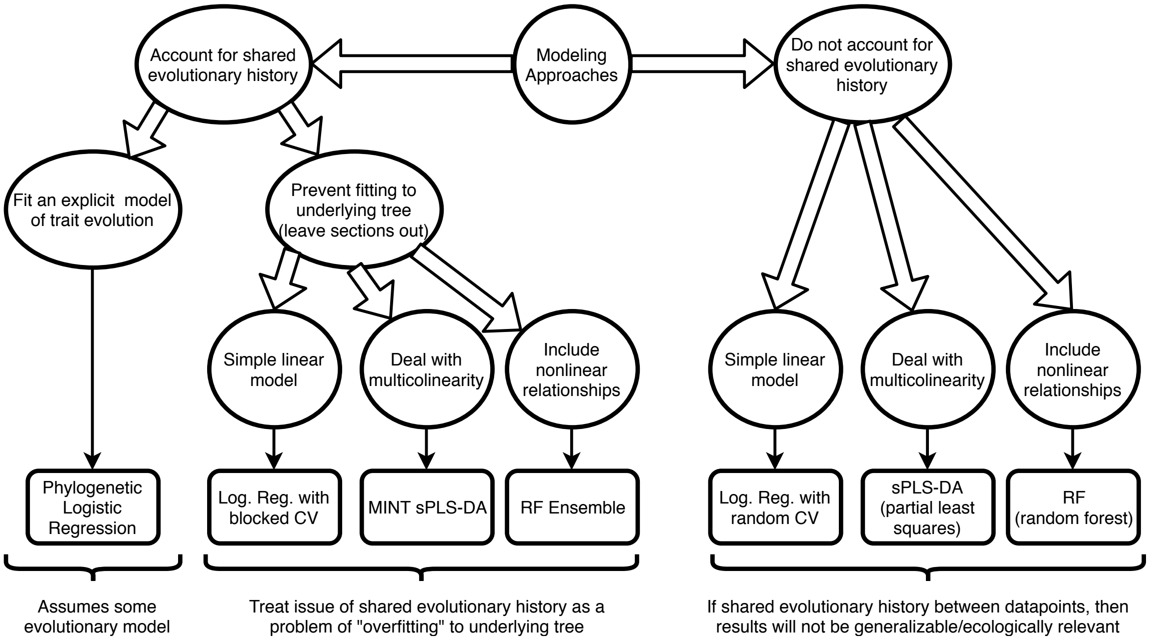

S18 Fig: Flowchart showing the decision-making process that would lead to the various modeling approaches used here. Major considerations for each approach are noted. See Methods for details on each approach.

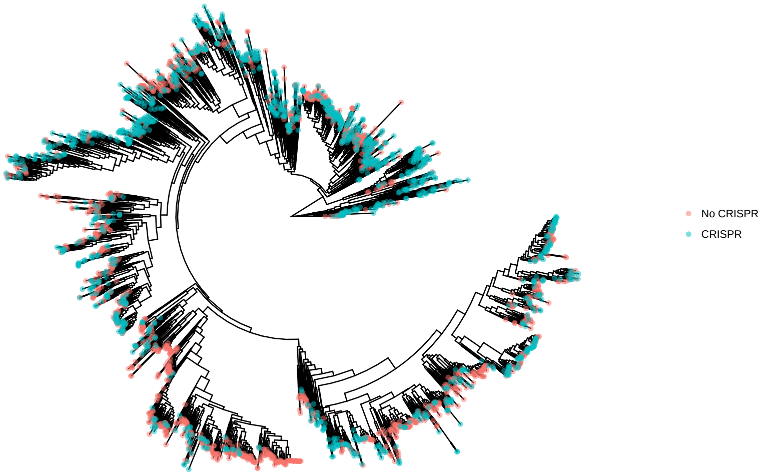

S19 Fig: Phylogeny generated from PhyloSift marker genes (as in Fig S16 Fig). Color indicates CRISPR incidence.

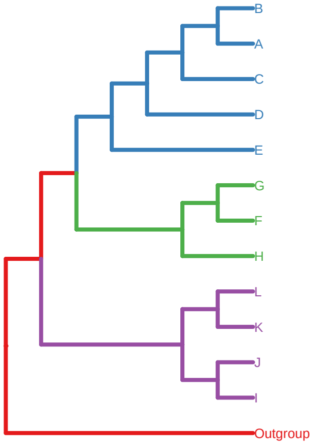

	Blocked Folds	Random Folds
Fold 1	A, B, C, D, E	D, H, K, L
Fold 2	F, G, H	A, B, G, I
Fold 3	I, J, K, L	C, E, F, J

S20 Fig: A conceptual example of the differences between blocked and random folds for cross validation. Cross validation (CV) relies on the assumption that folds are independent from one another, but when species share an evolutionary history this assumption is violated. By instead choosing folds based on phylogenetic groups that have diverged from each other sufficiently far in the past, we can better avoid the inclusion of phylogenetic signal in our model fit. In other words, in blocked CV we attempt to choose "evolutionarily independent" folds.

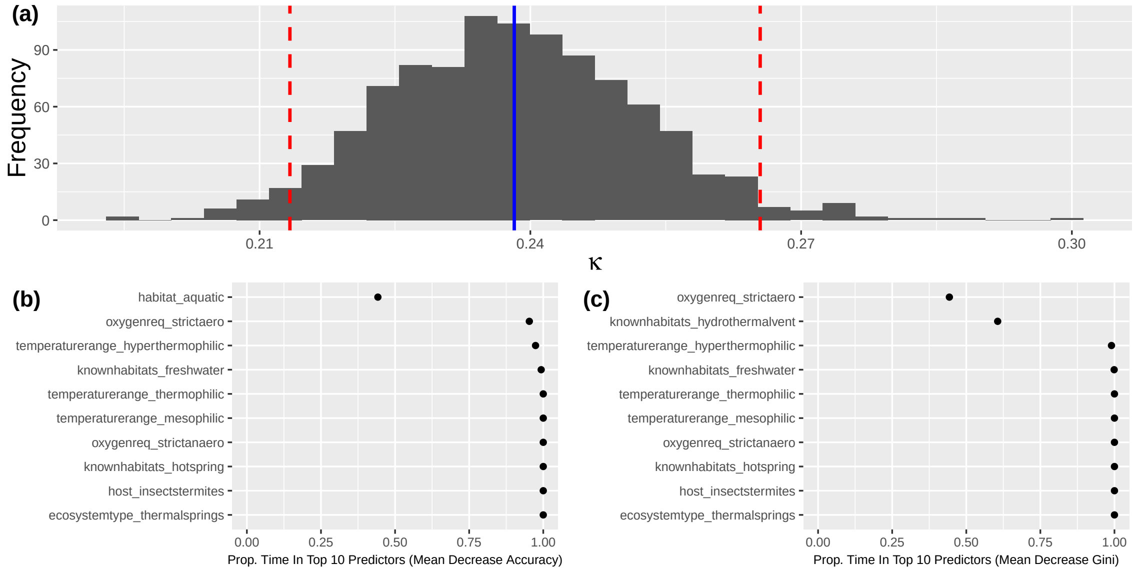

S21 Fig: Resampling genomes has little effect on our overall outcome. (a) Distribution of κ values for 1000 RF models built with resampled datasets. Mean (blue) and 95% CIs (red) indicated with vertical lines. (b-c) The proportion of resampled datasets for which each predictor fell within the set of top 10 predictors based on variable importance scores.

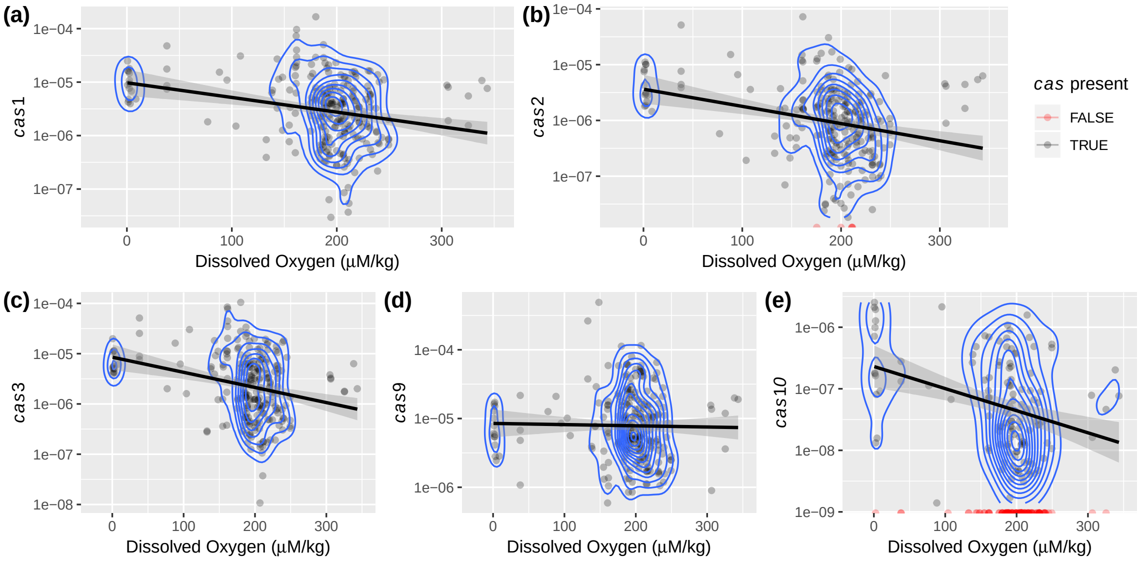

S22 Fig: Functional profiles for *cas* genes from Tara Oceans Project with corresponding oxygen metadata. Values for each *cas* gene shown are the coverage mapping to that orthologous group normalized by the total coverage in the metagenome. Zero values for coverage plotted along x-axis in red (since data plotted on log scale). Trend-lines plotted on log transformed data for ease of interpretation.
